# 3-(2-Amino-1,3-thia­zol-4-yl)-6-chloro-2*H*-chromen-2-one

**DOI:** 10.1107/S1600536809048247

**Published:** 2009-11-18

**Authors:** Deepak Chopra, A. R. Choudhury, K. N. Venugopala, Thavendran Govender, Hendrik G. Kruger, Glenn E. M. Maguire, T. N. Guru Row

**Affiliations:** a Department of Chemistry, Indian Institute of Science Education and Research, Bhopal 462 023, India; b Chemistry Group, Birla Institute of Technology and science, Pilani, Pilani, 333 031, Rajasthan, India; c School of Chemistry, University of Kwazulu-Natal, Durban 4000, South Africa; d School of Pharmacy and ­Pharmacology, University of Kwazulu-Natal, Durban 4000, South Africa; e Solid State and Structural Chemistry Unit, Indian Institute of Science, Bangalore 560 012, Karnataka, India

## Abstract

The title compound, C_12_H_7_ClN_2_O_2_S, crystallizes with two mol­ecules in the asymmetric unit. The mol­ecular conformation is roughly planar for both these mol­ecules with maximum deviations of 0.177 (3) and 0.076 (4) Å from their respective mean planes. In the crystal, strong N—H⋯N and weak but highly directional C—H⋯O hydrogen bonds provide the links between the mol­ecules. The structure is further stabilised by aromatic π–π stacking inter­actions with centroid–centroid distances in the range 3.650 (3)–3.960 (3) Å.

## Related literature

For applications of coumarin compounds in photochemistry, see: Vishnumurthy *et al.* (2001 ▶). For their roles as dyes, laser dyes and in probing ultrafast solvation effects see: Morris & Rusell (1971 ▶); Khalfan *et al.*, (1987 ▶); Maroncelli & Fleming (1987 ▶). For graph set motifs, see: Bernstein *et al.* (1995 ▶). For the synthesis of the title compound, see: Venugopal *et al.* (2004 ▶). For related structures see: Vishnumurthy *et al.* (2001 ▶).
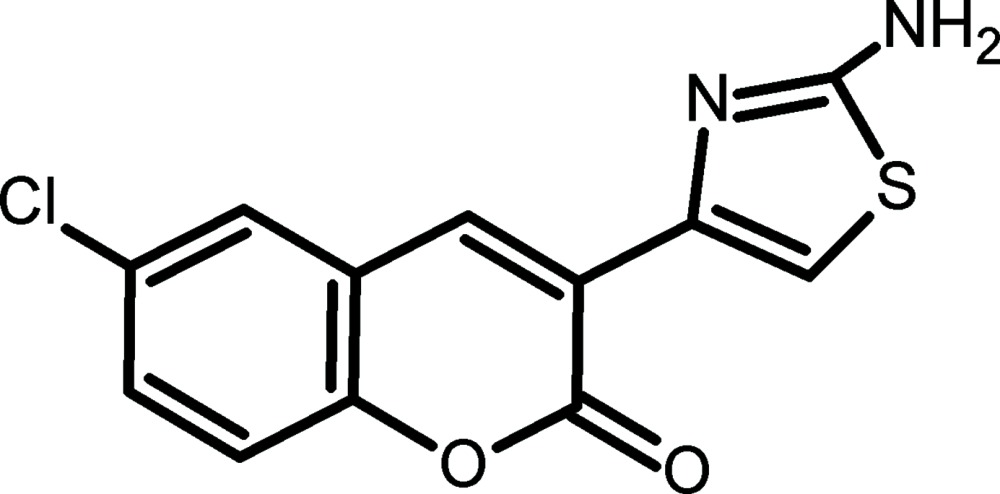


## Experimental

### 

#### Crystal data

C_12_H_7_ClN_2_O_2_S
*M* *_r_* = 278.72
Monoclinic, 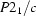

*a* = 12.494 (8) Å
*b* = 7.350 (5) Å
*c* = 25.013 (15) Å
β = 98.156 (12)°
*V* = 2274 (3) Å^3^
*Z* = 8
Mo *K*α radiation
μ = 0.51 mm^−1^
*T* = 290 K
0.20 × 0.10 × 0.02 mm

#### Data collection

Bruker SMART CCD area detector diffractometer
Absorption correction: multi-scan (*SADABS*; Sheldrick (1996 ▶) *T*_min_ = 0.885, *T*_max_ = 0.990
16106 measured reflections
4165 independent reflections
2561 reflections with *I* > 2σ(*I*)
*R*_int_ = 0.054

#### Refinement

*R*[*F* ^2^ > 2σ(*F* ^2^)] = 0.054
*wR*(*F* ^2^) = 0.118
*S* = 1.01
4165 reflections
325 parameters
H-atom parameters constrained
Δρ_max_ = 0.28 e Å^−3^
Δρ_min_ = −0.27 e Å^−3^

### 

Data collection: *SMART* (Bruker, 2000 ▶); cell refinement: *SAINT* (Bruker, 2000 ▶); data reduction: *SAINT*; program(s) used to solve structure: *SHELXS97* (Sheldrick, 2008 ▶); program(s) used to refine structure: *SHELXL97* (Sheldrick, 2008 ▶); molecular graphics: *ORTEP-3 for Windows* (Farrugia, 1997 ▶) and *CAMERON* (Watkin *et al.*, 1993 ▶); software used to prepare material for publication: *PLATON* (Spek, 2009 ▶).

## Supplementary Material

Crystal structure: contains datablocks global, I. DOI: 10.1107/S1600536809048247/sj2672sup1.cif

Structure factors: contains datablocks I. DOI: 10.1107/S1600536809048247/sj2672Isup2.hkl

Additional supplementary materials:  crystallographic information; 3D view; checkCIF report

## Figures and Tables

**Table 1:** Hydrogen-bond geometry (Å, °)

*D*—H⋯*A*	*D*—H	H⋯*A*	*D*⋯*A*	*D*—H⋯*A*
N2—H2*A*⋯N3^i^	0.86	2.31	3.124 (4)	158
N4—H4*A*⋯N1^ii^	0.86	2.27	3.116 (4)	168
C7—H7⋯O2^iii^	0.93	2.54	3.387 (4)	152

Symmetry codes: (i) 
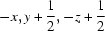
; (ii) 
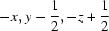
; (iii) 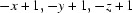.

**Fig. 1.**
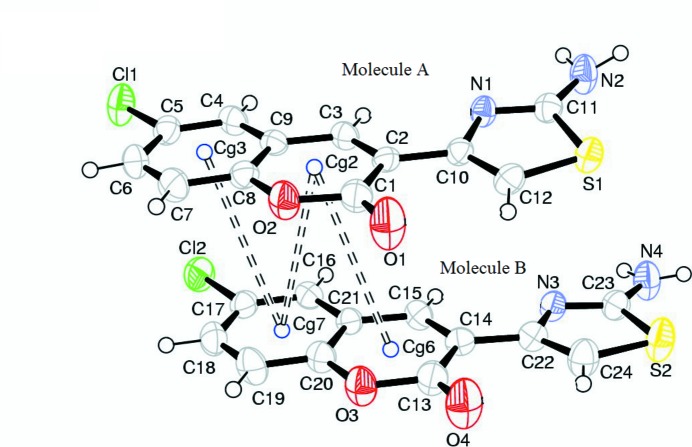
: The structure of the title compound drawn with 50% ellipsoidal probability showing the two molecules in the asymmetric unit (Molecules A and B). The dotted lines indicate inter-molecular centroid···centroid interactions. Cg2, Cg3, Cg6 and Cg7 are the centroids of the pyranone ring O2/C9 and the phenyl ring C4/C9 in Molecule A, and the pyranone ring O3/C15 and phenyl ring C16/C21 in Molecule B.

**Fig. 2.**
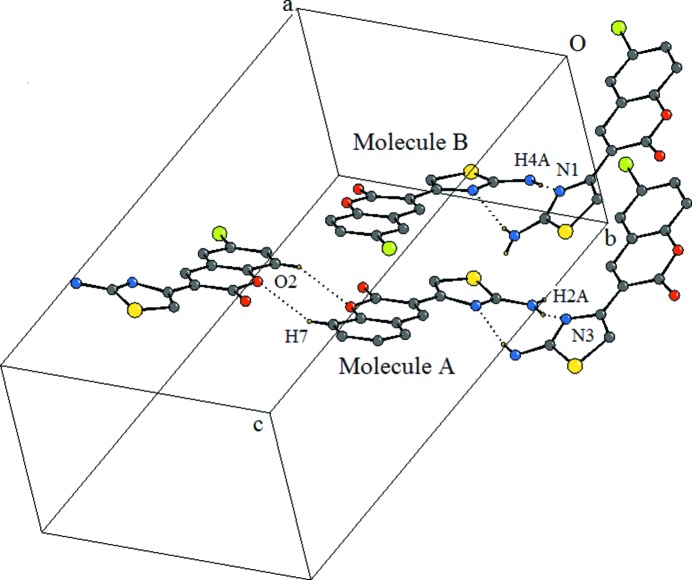
: Packing of the title compound highlighting the C—H···O dimers and N—H···N heterodimeric units. Only participating H atoms have been shown, others have been omitted for clarity.

## supplementary crystallographic information

### Comment

Coumarins are an important class of organic compounds and have been extensively studied.Such molecules of vast structural diversity find useful applications in several areas of synthetic chemistry, medicinal chemistry and photochemistry. The formation of [2 + 2] cycloaddition products upon irradiation (Vishnumurthy *et al.*,2001) of coumarin and its derivatives has contributed immensely to the area of solid-state chemistry. Several substituted coumarin derivatives find applications in the dye industry (Morris & Rusell, 1971) and in the area of LASER dyes (Khalfan *et al.*, 1987) based on the fact that such compounds show state dependent variations in their static dipole moments. These have also been used to probe ultrafast solvation effects (Maroncelli & Fleming, 1987). The geometry and molecular packing patterns of several coumarin derivatives have been studied to evaluate the features of non-covalent interactions (Vishnumurthy *et al.*, 2001). Against this background, and to obtain more information on such compounds the solid-state structure of the title compound is reported here.

In the title compound, we have a chloro substituted coumarin ring crystallizing in monoclinic centrosymmetric space group with two unique molecules in the asymmetric unit [(A) and (B)]. Both the molecules are essentially planar with the dihedral angles between the least squares planes passing through the coumarin ring and thiazoyl ring being 9.1 (1) and 4.9 (1)Å in A and B respectively. The largest displacement is observed for the atom C11 being -0.020 (3)Å for molecule A and atom C13 being -0.041 (4)Å for molecule B from the weighted least squares planes through C1/O2 and C13/O3 respectively. Aromatic π···π stacking interactions are found with distances *Cg*2···*Cg*6 = 3.942 (3) Å, *Cg*2···*Cg*7 = 3.650 (3) Å, and *Cg*3···*Cg*7 = 3.960 (3)Å between the molecules A and B. *Cg*2, *Cg*3, *Cg*6 and *Cg*7 are the centroids of the six-membered rings O2/C8, C4/C9, O3/C20 and C16/C21 (Figure 1). Molecules A are linked by alternating C—H···O interactions (involving H7 and O2) forming *R*^2^2(8) ring dimers [Bernstein *et al.*, 1995]. N—H···N hydrogen bonds form hetero-dimeric motifs linking A and B molecules. (Figure 2, Table 1). Thus the supramolecular assembly is built up by an interplay of strong N—H···N, weak C—H···O and π···π van der Waals interactions. A short Cl···S contact of distance 3.532 (2)Å ( symmetry code: x, -y+1+1/2, z-1/2) is also present in the crystal lattice

### Experimental

The compounds were synthesized in accordance with the procedure reported in the literature (Venugopal *et al.*, 2004). Single crystals of the compound were grown from chloroform:methanol (1:1) by slow evaporation at 275–277 K.

### Refinement

All H-atoms were positioned geometrically and refined using a riding model with d(C-H) = 0.93Å, U_iso_=1.2U_eq_ (C) for aromatic and 0.86Å, U_iso_ = 1.2U_eq_ (N) for the NH atoms.

### Crystal data

**Table:**

C_12_H_7_ClN_2_O_2_S	*F*(000) = 1136
*M**_r_* = 278.72	*D*_x_ = 1.628 Mg m^−^^3^
Monoclinic, *P*2_1_/*c*	Mo *K*α radiation, λ = 0.71073 Å
Hall symbol: -P 2ybc	Cell parameters from 845 reflections
*a* = 12.494 (8) Å	θ = 1.6–25.8°
*b* = 7.350 (5) Å	µ = 0.51 mm^−^^1^
*c* = 25.013 (15) Å	*T* = 290 K
β = 98.156 (12)°	Plate, yellow
*V* = 2274 (3) Å^3^	0.20 × 0.10 × 0.02 mm
*Z* = 8	

### Data collection

**Table:**

Bruker SMART CCD area detector diffractometer	4165 independent reflections
Radiation source: fine-focus sealed tube	2561 reflections with *I* > 2σ(*I*)
graphite	*R*_int_ = 0.054
φ and ω scans	θ_max_ = 25.4°, θ_min_ = 1.6°
Absorption correction: multi-scan (*SADABS*; Sheldrick (1996)	*h* = −15→15
*T*_min_ = 0.885, *T*_max_ = 0.990	*k* = −8→8
16106 measured reflections	*l* = −29→30

### Refinement

**Table:**

Refinement on *F*^2^	Primary atom site location: structure-invariant direct methods
Least-squares matrix: full	Secondary atom site location: difference Fourier map
*R*[*F*^2^ > 2σ(*F*^2^)] = 0.054	Hydrogen site location: inferred from neighbouring sites
*wR*(*F*^2^) = 0.118	H-atom parameters constrained
*S* = 1.01	*w* = 1/[σ^2^(*F*_o_^2^) + (0.0493*P*)^2^] where *P* = (*F*_o_^2^ + 2*F*_c_^2^)/3
4165 reflections	(Δ/σ)_max_ < 0.001
325 parameters	Δρ_max_ = 0.28 e Å^−^^3^
0 restraints	Δρ_min_ = −0.27 e Å^−^^3^

### Special details

**Table:**

Geometry. All e.s.d.'s (except the e.s.d. in the dihedral angle between two l.s. planes) are estimated using the full covariance matrix. The cell e.s.d.'s are taken into account individually in the estimation of e.s.d.'s in distances, angles and torsion angles; correlations between e.s.d.'s in cell parameters are only used when they are defined by crystal symmetry. An approximate (isotropic) treatment of cell e.s.d.'s is used for estimating e.s.d.'s involving l.s. planes.
Refinement. Refinement of *F*^2^ against ALL reflections. The weighted *R*-factor *wR* and goodness of fit *S* are based on *F*^2^, conventional *R*-factors *R* are based on *F*, with *F* set to zero for negative *F*^2^. The threshold expression of *F*^2^ > σ(*F*^2^) is used only for calculating *R*-factors(gt) *etc*. and is not relevant to the choice of reflections for refinement. *R*-factors based on *F*^2^ are statistically about twice as large as those based on *F*, and *R*- factors based on ALL data will be even larger.

### Fractional atomic coordinates and isotropic or equivalent isotropic displacement parameters (Å^2^)

**Table:**

	*x*	*y*	*z*	*U*_iso_*/*U*_eq_	
S1	0.23200 (7)	0.90698 (12)	0.22923 (3)	0.0415 (2)	
S2	0.28374 (8)	0.49152 (15)	0.13304 (4)	0.0563 (3)	
Cl1	0.07808 (7)	0.52598 (14)	0.60106 (3)	0.0559 (3)	
Cl2	0.12399 (7)	0.11642 (12)	0.50115 (3)	0.0533 (3)	
O1	0.44938 (19)	0.7077 (4)	0.37710 (10)	0.0724 (8)	
O2	0.39831 (17)	0.6431 (3)	0.45510 (9)	0.0475 (6)	
O3	0.45453 (17)	0.2376 (3)	0.36083 (9)	0.0485 (6)	
O4	0.50908 (19)	0.3322 (4)	0.28593 (10)	0.0684 (8)	
N1	0.12697 (19)	0.8694 (3)	0.31039 (10)	0.0338 (6)	
N2	0.0231 (3)	0.9983 (5)	0.23312 (13)	0.0502 (9)	
N3	0.1780 (2)	0.4062 (3)	0.21072 (10)	0.0365 (6)	
N4	0.0660 (3)	0.4837 (5)	0.13067 (14)	0.0530 (9)	
C1	0.3740 (3)	0.6983 (5)	0.40182 (14)	0.0436 (9)	
C2	0.2602 (2)	0.7400 (4)	0.38192 (12)	0.0308 (7)	
C3	0.1857 (3)	0.7177 (4)	0.41557 (13)	0.0332 (8)	
C4	0.1379 (3)	0.6270 (4)	0.50590 (13)	0.0366 (8)	
C5	0.1731 (3)	0.5640 (4)	0.55710 (13)	0.0370 (8)	
C6	0.2809 (3)	0.5279 (5)	0.57507 (15)	0.0444 (9)	
C7	0.3557 (3)	0.5552 (5)	0.54048 (14)	0.0436 (9)	
C8	0.3206 (2)	0.6182 (4)	0.48857 (13)	0.0353 (8)	
C9	0.2130 (2)	0.6547 (4)	0.47006 (12)	0.0319 (7)	
C10	0.2323 (2)	0.8062 (4)	0.32644 (12)	0.0317 (7)	
C11	0.1171 (3)	0.9292 (4)	0.26057 (13)	0.0352 (8)	
C12	0.2989 (3)	0.8191 (4)	0.28810 (13)	0.0388 (8)	
C13	0.4323 (3)	0.3045 (5)	0.30836 (14)	0.0443 (9)	
C14	0.3174 (2)	0.3330 (4)	0.28654 (12)	0.0340 (8)	
C15	0.2416 (3)	0.3064 (4)	0.31904 (13)	0.0346 (8)	
C16	0.1909 (3)	0.2176 (4)	0.40798 (13)	0.0354 (8)	
C17	0.2229 (3)	0.1554 (4)	0.45952 (13)	0.0378 (8)	
C18	0.3301 (3)	0.1209 (5)	0.47895 (14)	0.0441 (9)	
C19	0.4068 (3)	0.1491 (5)	0.44523 (15)	0.0484 (10)	
C20	0.3756 (3)	0.2112 (4)	0.39311 (13)	0.0384 (8)	
C21	0.2678 (2)	0.2457 (4)	0.37355 (12)	0.0315 (7)	
C22	0.2884 (2)	0.3895 (4)	0.23001 (13)	0.0357 (8)	
C23	0.1654 (3)	0.4575 (4)	0.16061 (13)	0.0375 (8)	
C24	0.3552 (3)	0.4296 (5)	0.19410 (14)	0.0487 (10)	
H2A	−0.0333	1.0075	0.2492	0.062*	
H2B	0.0214	1.0376	0.2008	0.062*	
H3	0.1138	0.7442	0.4028	0.039*	
H4	0.0648	0.6505	0.4950	0.044*	
H4A	0.0083	0.4664	0.1450	0.063*	
H4B	0.0614	0.5185	0.0976	0.063*	
H6	0.3028	0.4849	0.6099	0.053*	
H7	0.4289	0.5323	0.5515	0.052*	
H12	0.3712	0.7846	0.2928	0.047*	
H15	0.1694	0.3273	0.3053	0.041*	
H16	0.1181	0.2406	0.3960	0.042*	
H18	0.3502	0.0795	0.5140	0.053*	
H19	0.4799	0.1262	0.4575	0.057*	
H24	0.4303	0.4239	0.2010	0.059*	

### Atomic displacement parameters (Å^2^)

**Table:**

	*U*^11^	*U*^22^	*U*^33^	*U*^12^	*U*^13^	*U*^23^
S1	0.0423 (5)	0.0528 (6)	0.0305 (5)	−0.0011 (4)	0.0088 (4)	0.0006 (4)
S2	0.0480 (6)	0.0865 (8)	0.0360 (5)	−0.0034 (5)	0.0115 (4)	0.0097 (5)
Cl1	0.0482 (6)	0.0858 (8)	0.0339 (5)	−0.0029 (5)	0.0069 (4)	0.0074 (5)
Cl2	0.0571 (6)	0.0611 (6)	0.0452 (6)	0.0043 (5)	0.0192 (5)	0.0086 (5)
N1	0.0300 (14)	0.0429 (17)	0.0278 (15)	−0.0001 (12)	0.0019 (12)	0.0017 (12)
N2	0.039 (2)	0.076 (3)	0.0348 (19)	0.0068 (18)	0.0018 (15)	0.0118 (18)
N3	0.0352 (16)	0.0448 (17)	0.0299 (16)	0.0003 (13)	0.0055 (12)	0.0012 (13)
N4	0.045 (2)	0.076 (3)	0.037 (2)	0.0023 (18)	0.0042 (17)	0.0137 (18)
O1	0.0354 (15)	0.132 (3)	0.0524 (17)	0.0247 (16)	0.0163 (13)	0.0260 (17)
O2	0.0306 (12)	0.0713 (17)	0.0400 (14)	0.0133 (12)	0.0030 (11)	0.0089 (13)
O3	0.0298 (13)	0.0725 (17)	0.0423 (15)	0.0043 (12)	0.0021 (11)	0.0100 (12)
O4	0.0332 (14)	0.118 (2)	0.0566 (17)	0.0055 (15)	0.0144 (13)	0.0211 (16)
C1	0.039 (2)	0.052 (2)	0.041 (2)	0.0098 (17)	0.0071 (17)	0.0030 (18)
C2	0.0300 (17)	0.0317 (19)	0.0310 (18)	0.0053 (14)	0.0054 (14)	−0.0028 (14)
C3	0.0269 (19)	0.0353 (19)	0.035 (2)	0.0018 (15)	−0.0019 (16)	−0.0002 (15)
C4	0.0264 (19)	0.040 (2)	0.040 (2)	0.0022 (15)	−0.0052 (16)	0.0005 (16)
C5	0.039 (2)	0.038 (2)	0.0329 (19)	−0.0025 (16)	0.0025 (15)	−0.0023 (15)
C6	0.047 (2)	0.051 (2)	0.032 (2)	0.0050 (18)	−0.0068 (17)	0.0015 (18)
C7	0.032 (2)	0.055 (2)	0.041 (2)	0.0079 (18)	−0.0040 (17)	0.0004 (18)
C8	0.0329 (18)	0.0380 (19)	0.0345 (19)	0.0044 (15)	0.0032 (15)	−0.0041 (16)
C9	0.0303 (18)	0.0296 (18)	0.0350 (19)	0.0034 (14)	0.0024 (15)	−0.0001 (14)
C10	0.0322 (18)	0.0339 (19)	0.0285 (18)	0.0001 (15)	0.0027 (14)	−0.0030 (15)
C11	0.0360 (19)	0.0357 (19)	0.0333 (19)	−0.0048 (15)	0.0025 (15)	−0.0029 (15)
C12	0.035 (2)	0.045 (2)	0.038 (2)	0.0007 (17)	0.0097 (16)	−0.0035 (16)
C13	0.035 (2)	0.058 (2)	0.039 (2)	0.0054 (17)	0.0053 (17)	0.0029 (18)
C14	0.0294 (18)	0.0353 (19)	0.0372 (19)	−0.0003 (14)	0.0044 (15)	−0.0022 (15)
C15	0.0269 (19)	0.039 (2)	0.036 (2)	−0.0015 (16)	−0.0037 (15)	0.0005 (16)
C16	0.034 (2)	0.034 (2)	0.038 (2)	0.0010 (16)	0.0022 (17)	0.0013 (16)
C17	0.046 (2)	0.0333 (19)	0.036 (2)	−0.0013 (16)	0.0108 (16)	−0.0002 (15)
C18	0.048 (2)	0.050 (2)	0.033 (2)	0.0004 (18)	−0.0014 (18)	0.0029 (18)
C19	0.030 (2)	0.064 (3)	0.048 (2)	0.0042 (18)	−0.0097 (18)	0.0080 (19)
C20	0.0322 (19)	0.043 (2)	0.038 (2)	−0.0008 (16)	0.0008 (15)	0.0002 (16)
C21	0.0293 (17)	0.0329 (19)	0.0313 (19)	0.0010 (14)	0.0006 (14)	−0.0019 (14)
C22	0.0324 (18)	0.038 (2)	0.037 (2)	−0.0023 (15)	0.0052 (15)	−0.0010 (16)
C23	0.037 (2)	0.040 (2)	0.034 (2)	−0.0023 (15)	0.0020 (16)	−0.0031 (16)
C24	0.040 (2)	0.065 (3)	0.042 (2)	−0.0030 (19)	0.0088 (18)	0.0055 (19)

### Geometric parameters (Å, °)

**Table:**

S1—C12	1.713 (4)	C13—C14	1.475 (4)
S1—C11	1.738 (3)	C7—C6	1.376 (5)
Cl2—C17	1.749 (3)	C7—H7	0.9300
Cl1—C5	1.750 (3)	C2—C10	1.464 (4)
S2—C24	1.714 (4)	C2—C1	1.470 (5)
S2—C23	1.736 (3)	C15—C14	1.347 (4)
C3—C2	1.349 (4)	C15—H15	0.9300
C3—C9	1.436 (4)	C22—C24	1.347 (4)
C3—H3	0.9300	C22—C14	1.467 (4)
O3—C20	1.371 (4)	C23—N4	1.369 (4)
O3—C13	1.392 (4)	C10—C12	1.364 (4)
O2—C8	1.381 (4)	C5—C4	1.375 (4)
O2—C1	1.385 (4)	C5—C6	1.388 (5)
N3—C23	1.298 (4)	C1—O1	1.200 (4)
N3—C22	1.402 (4)	C18—C19	1.381 (5)
C8—C9	1.385 (4)	C18—C17	1.385 (5)
C8—C7	1.387 (4)	C18—H18	0.9300
C16—C17	1.372 (4)	C11—N2	1.369 (4)
C16—C21	1.395 (4)	C6—H6	0.9300
C16—H16	0.9300	C4—H4	0.9300
C21—C20	1.392 (4)	C24—H24	0.9300
C21—C15	1.429 (4)	C19—H19	0.9300
C9—C4	1.402 (4)	C12—H12	0.9300
C20—C19	1.385 (4)	N4—H4A	0.8600
N1—C11	1.311 (4)	N4—H4B	0.8600
N1—C10	1.401 (4)	N2—H2A	0.8600
C13—O4	1.196 (4)	N2—H2B	0.8600
			
C12—S1—C11	89.03 (16)	C12—C10—N1	114.5 (3)
C24—S2—C23	88.61 (17)	C12—C10—C2	127.5 (3)
C2—C3—C9	122.6 (3)	N1—C10—C2	118.0 (3)
C2—C3—H3	118.7	C15—C14—C22	121.5 (3)
C9—C3—H3	118.7	C15—C14—C13	119.1 (3)
C20—O3—C13	122.8 (3)	C22—C14—C13	119.3 (3)
C8—O2—C1	123.1 (3)	C4—C5—C6	122.3 (3)
C23—N3—C22	109.8 (3)	C4—C5—Cl1	118.9 (3)
O2—C8—C9	120.2 (3)	C6—C5—Cl1	118.8 (3)
O2—C8—C7	117.0 (3)	O1—C1—O2	115.5 (3)
C9—C8—C7	122.8 (3)	O1—C1—C2	127.3 (3)
C17—C16—C21	119.6 (3)	O2—C1—C2	117.1 (3)
C17—C16—H16	120.2	C19—C18—C17	119.0 (3)
C21—C16—H16	120.2	C19—C18—H18	120.5
C20—C21—C16	118.3 (3)	C17—C18—H18	120.5
C20—C21—C15	118.2 (3)	N1—C11—N2	123.8 (3)
C16—C21—C15	123.4 (3)	N1—C11—S1	115.1 (2)
C8—C9—C4	117.7 (3)	N2—C11—S1	121.0 (3)
C8—C9—C3	118.0 (3)	C16—C17—C18	122.0 (3)
C4—C9—C3	124.2 (3)	C16—C17—Cl2	118.5 (3)
O3—C20—C19	118.0 (3)	C18—C17—Cl2	119.5 (3)
O3—C20—C21	120.4 (3)	C7—C6—C5	119.0 (3)
C19—C20—C21	121.6 (3)	C7—C6—H6	120.5
C11—N1—C10	110.0 (3)	C5—C6—H6	120.5
O4—C13—O3	115.8 (3)	C5—C4—C9	119.3 (3)
O4—C13—C14	127.3 (3)	C5—C4—H4	120.4
O3—C13—C14	116.9 (3)	C9—C4—H4	120.4
C6—C7—C8	118.9 (3)	C22—C24—S2	111.1 (3)
C6—C7—H7	120.5	C22—C24—H24	124.4
C8—C7—H7	120.5	S2—C24—H24	124.4
C3—C2—C10	122.6 (3)	C18—C19—C20	119.9 (3)
C3—C2—C1	118.9 (3)	C18—C19—H19	120.2
C10—C2—C1	118.5 (3)	C20—C19—H19	120.2
C14—C15—C21	122.3 (3)	C10—C12—S1	111.2 (2)
C14—C15—H15	118.8	C10—C12—H12	124.4
C21—C15—H15	118.8	S1—C12—H12	124.4
C24—C22—N3	114.9 (3)	C23—N4—H4A	120.0
C24—C22—C14	128.1 (3)	C23—N4—H4B	120.0
N3—C22—C14	117.0 (3)	H4A—N4—H4B	120.0
N3—C23—N4	123.2 (3)	C11—N2—H2A	120.0
N3—C23—S2	115.5 (3)	C11—N2—H2B	120.0
N4—C23—S2	121.3 (3)	H2A—N2—H2B	120.0

### Hydrogen-bond geometry (Å, °)

**Table:**

*D*—H···*A*	*D*—H	H···*A*	*D*···*A*	*D*—H···*A*
N2—H2A···N3^i^	0.86	2.31	3.124 (4)	158
N4—H4A···N1^ii^	0.86	2.27	3.116 (4)	168
C7—H7···O2^iii^	0.93	2.54	3.387 (4)	152

Symmetry codes: (i) −*x*, *y*+1/2, −*z*+1/2; (ii) −*x*, *y*−1/2, −*z*+1/2; (iii) −*x*+1, −*y*+1, −*z*+1.
